# The use of quantitative clinical pharmacology approaches to support moxidectin dosing recommendations in lactation

**DOI:** 10.1371/journal.pntd.0012351

**Published:** 2024-08-05

**Authors:** Nolan D. Wood, Danelle Smith, Sally A. Kinrade, Mark T. Sullivan, Craig R. Rayner, David Wesche, Kashyap Patel, Karen Rowland-Yeo

**Affiliations:** 1 Certara, Princeton, New Jersey, United States of America; 2 Medicines Development for Global Health, Southbank, Victoria, Australia; 3 Kirby Institute, UNSW Sydney, Sydney, New South Wales, Australia; McGill University, CANADA

## Abstract

Moxidectin is approved by the US Food and Drug Administration (US FDA) for the treatment of onchocerciasis (river-blindness) due to *Onchocerca volvulus* in patients aged 12 years and older. In onchocerciasis-endemic areas, mass drug administration (MDA) programs with ivermectin, with or without vector control, aim to control the disease, reduce morbidity, interrupt transmission, and more recently, achieve elimination. Moxidectin has the potential to be used in MDA programs. In countries where onchocerciasis is endemic, infants are often breastfed up to the age of 2 years, suggesting that some women are likely to be lactating during such periodic MDA programs. Quantitative analyses of non-clinical and clinical data using non-compartmental analysis and population based pharmacokinetic (popPK) modeling as well as physiologically based pharmacokinetic modeling (PBPK) were performed to determine the amount of moxidectin excreted in breast milk and subsequent exposures in the infant. The results of the analyses were similar. Concentrations of moxidectin in breast milk followed a similar pattern to those in plasma, with maximum concentrations occurring approximately 4 hours after dosing followed by a rapid decline in both breast milk and plasma. As early as two days after dosing, concentrations of moxidectin in breast milk were below the threshold for acceptable daily intake levels established by the European Medicines Agency (EMA) and FDA for secondary exposures from veterinary use, and below the WHO recommended relative infant dose (RID) safety threshold. The analyses were conducted to support prescribers and policy makers on dosing recommendations for moxidectin in lactation.

## Author summary

Moxidectin is approved to treat onchocerciasis or “river-blindness”. For years, mass drug administration programs (MDA) involving at least annual treatment of entire populations within a geographical area have been used as a strategy to control onchocerciasis. The potential treatment population therefore includes pregnant and breast-feeding women. Guidance on the safe and effective use of medicines in these populations is needed but is often insufficient. In this study, we describe two methods to determine the amount of moxidectin administered to breastfeeding infants via breastmilk. Both methods used data from a clinical study in which moxidectin levels were measured in the blood and breast milk of lactating women. In an empirical analysis, the amount of moxidectin in a breast-fed infant was inferred based on observed blood and breast milk levels in the clinical study. Using a physiologically based pharmacokinetic (PBPK) model approach, which considers all prior information on the drug and the physiological variability involved, direct assessments of the amount of drug in the infant were estimated. Both methods established the likely time-course and level of moxidectin exposure in infants via breast milk, highlighting the benefit of an integrated approach to generate dosing recommendations supporting the use of moxidectin in breastfeeding women.

## Introduction

Onchocerciasis (river blindness) is a neglected tropical disease (NTD) caused by the filarial nematode *Onchocerca (O*.*) volvulus* and is endemic in many regions of sub-Saharan Africa. The disease is caused by the host response to the life and death of microfilariae in the skin and eyes. The parasite is transmitted to humans by the bites of infected blackflies of the genus *Simulium* and is the second leading infectious cause of blindness globally. In 2017, it was estimated that there were 20.9 million *O*. *volvulus* infections worldwide [1].

Ivermectin, available as tablets for oral administration (Stromectol or Mectizan) [2], is a macrocyclic lactone of the avermectin class, and has been recommended for the treatment of onchocerciasis for many years [3,4]. Since 1988, The World Health Organization (WHO) has promoted the distribution of ivermectin in mass drug administration (MDA) programs to control the disease by reducing microfilaridermia, preventing blindness secondary to the presence of microfilariae in the eye, and to ameliorate disease symptoms such as intense pruritis. The goals of treatment are to reduce morbidity, interrupt transmission, and, more recently, to achieve onchocerciasis elimination [5]. Ivermectin is distributed at least annually in areas with ongoing transmission to all eligible community members, with the aim of achieving at least 80% therapeutic coverage in each treatment round [6,7].

Moxidectin, a macrocyclic lactone of the milbemycin class, has recently been approved by the US Food and Drug Administration (FDA) as a single oral dose of 8 mg for the treatment of onchocerciasis in people aged 12 years and older [8]. A review of the chemistry, pharmacology and clinical studies providing evidence on the safety, tolerability and efficacy of moxidectin in the treatment of human onchocerciasis has been published [9]. In addition, the pharmacokinetics of orally administered moxidectin following administration to adults with *O*. *volvulus* infection have also recently been published [10]. Compared to a standard dose of ivermectin, the terminal elimination half-life and the resultant reduction in microfilaridermia are substantially longer following a single oral dose of 8 mg moxidectin, offering potential logistical and therapeutic advantages for MDA treatment programs.

Neglected tropical diseases (NTDs) are prevalent in low- and middle-income countries, affecting some of the poorest populations in the world and often disproportionately affect pregnant women, nursing mothers and children [11,12]. These vulnerable groups are frequently underrepresented in clinical trial populations, and thus access to approved medications also lags. Furthermore, in countries where onchocerciasis is endemic [13], infants are routinely breastfed up to the age of 2 years and it is estimated that at any time approximately 8% of the population are likely to be pregnant or breast-feeding women. To exclude this substantial proportion of the population in control programs means that they are not only deprived of the potential benefits of treatment, but they may also remain a reservoir of infection within their communities [14]. Guidance on the effective use of medicines to treat onchocerciasis and other NTDs in these populations is needed, but often lacking [15].

The US approved product label for ivermectin (Stromectol) states that “Ivermectin should not be used during pregnancy since safety in pregnancy has not been established” [2]. In addition, the US approved product label states “Stromectol is excreted in human milk in low concentrations. Treatment of mothers who intend to breast-feed should only be undertaken when the risk of delayed treatment to the mother outweighs the possible risk to the newborn.” In other jurisdictions (e.g. Europe, Canada and Australia) it is recommended that ivermectin should only be given to nursing mothers if the benefit to the mother outweighs the potential risk to the breast-fed infant, and treatment of mothers who intend to breast feed their infants should be delayed until at least 1 week after birth of the child [16]. The WHO also currently recommends that ivermectin should not be administered to lactating women in the first week after the child’s birth [5].

Data on the excretion of ivermectin in human breastmilk following oral administration are limited and no prospective, regulatory-standard clinical trials have been performed. In four lactating women who received a single oral dose of 150 microgram (μg) per kilogram (kg), ivermectin was detected in milk within 1 hour after dosing and up to 72 hours post dose. Average peak milk levels were 15 ng/mL (range 11 to 21 ng/mL) and it was estimated that an exclusively breastfed infant weighing 3.5 kg would receive an average dose of approximately 5.15 μg of ivermectin (1.47 μg/kg), equivalent to 0.98% of the weight-adjusted maternal dose [17,18]. In a separate study in a woman treated with a single oral dose of 200 μg/kg ivermectin for *Strongyloides stercoralis*, peak milk levels of 20.8 ng/mL were observed 6 hours after dosing with an average concentration of 9.26 ng/mL over a 24-hour period equating to approximately 1.39 μg/kg, or 0.70% of the weight-adjusted maternal dose in an exclusively breastfed infant [19]. Accurate assessments of the amount of drug excreted in breast milk, and subsequent exposure to the infant would be beneficial regarding appropriate dosing recommendations in this population, and specifically when it would be considered acceptable to administer treatment to breastfeeding mothers participating in MDA programs. Application of quantitative clinical pharmacology techniques represents a method by which data can be assessed to enable informed decisions supporting the safe and effective use of medications in these populations.

For moxidectin, a dedicated regulatory-standard breast milk excretion study has been conducted. The pharmacokinetics of a single dose of 8 mg moxidectin, the recommended therapeutic dose, were studied in 12 healthy lactating women, aged 23–38 years, weighing 54–79 kg, all more than 5 months postpartum. Maternal plasma samples were collected for 90 days, and complete milk collection was performed for approximately 28 days [20]. The mean (and standard deviation, SD) maximum plasma concentration (C_max_) and area under the plasma concentration-time curve (AUC) were 87 ± 25 ng/mL and 4046 ± 1796 ng.h/mL, respectively. The mean drug-in-milk to plasma ratio, assessed as AUCmilk/AUCplasma, was 1.77± 0.66. The estimated mean (±SD) total infant dose, assuming the infants would consume all the breast milk collected during the study, and assuming 100% bioavailability of the amount excreted, was 56 ± 24 μg which represents approximately 0.7% (± 0.30%) of the maternal dose.

The estimated milk to plasma ratio and the estimated total infant dose were included in the initial approved US prescribing information for moxidectin in 2018 [21], along with the following statements: “There are no data on the effects of Moxidectin Tablets on the breast-fed infant or milk production. The developmental and health benefits of breastfeeding should be considered along with the mother’s clinical need for Moxidectin Tablets and any potential adverse effects on the breastfed child from Moxidectin Tablets or from the underlying maternal condition.” Thus, no specific guidance was provided with respect to dosing recommendations for breastfeeding mothers.

Since the initial approval of moxidectin, additional non-clinical toxicology data have been generated that further informs maternal/foetal risk. These additional data have now been analysed in conjunction with the existing clinical data using quantitative clinical pharmacology approaches. The purpose of these analyses was to establish the likely time-course and level of exposure of infants to moxidectin via breast milk in order to provide guidance for prescribers and policy makers in making appropriate decisions for its use in breastfeeding women.

## Methods

Empirical analyses were conducted based on the existing exposure data in plasma and milk from the regulatory-standard clinical study conducted in healthy lactating women and the subsequent estimates of the relative exposures to the infant [20]. Estimated systemic exposures of moxidectin in onchocerciasis patients were also derived using an established population pharmacokinetic (pop PK) model [22]. These data were then compared to the exposure data generated in the additional non-clinical toxicology studies. The data were also reviewed in the context of suggested acceptable daily intake levels of incidental exposure of moxidectin established for moxidectin use in food producing animals in both the US and the EU [23,24].

Importantly, to estimate the time-course of moxidectin exposures in a breastfed infant [25,26] and to allow comparisons to exposure data generated in the non-clinical toxicology studies, a fully quantitative approach, including the development of a physiologically-based pharmacokinetic (PBPK) model was also performed. PBPK models marry the complex interplay of physiological parameters with drug characteristics, thus providing a mechanistic approach to predict the PK of drugs in different populations, including pediatrics [27]. Furthermore, pediatric PBPK models integrate additional information regarding organ development and ontogeny of pathways involved in drug disposition[28] and are frequently used to predict exposures in different age groups, including neonates, relative to adults [29,30,31]. Given the complex disposition and tissue distribution of moxidectin [22], PBPK modeling was used to predict the plasma exposure of moxidectin in neonates and infants up to the age of 6 months taking into account the time-variant physiology over the period of breastfeeding.

### Method 1: Empirical model approach

Mean plasma concentrations at 24 and 48 hours post dose, and mean AUC at 1, 2, 3, 4, 8 and 12 weeks after a single oral dose of 8 mg moxidectin were derived by non-compartmental analysis (NCA) of the plasma concentration-time data from the regulatory-standard breast milk excretion study [20] using Phoenix WinNonlin (Version 8.1). In addition, estimates of C_max_ and plasma concentrations on Days 3, 7 and 30 following a single oral dose of 8 mg moxidectin to a typical healthy female subject and a typical female onchocerciasis patient were derived using an established pop PK model in NONMEM (version 7.3).

The NCA derived exposure data from the regulatory-standard clinical study conducted in healthy lactating women, and estimated exposures derived using the population PK model for a single oral dose of 8 mg moxidectin were compared to exposures from non-clinical animal toxicology studies to determine relative safety margins at each of the various timepoints post dose. The mean absolute infant dose of moxidectin was also estimated based on the amount excreted in breast milk in the regulatory-standard clinical study. Estimates of absolute infant dose were determined assuming breastfeeding commenced at the time as moxidectin administration, at 24 hours and at 1 week after dosing.

### Method 2: PBPK modeling

The Simcyp (V20.1) population-based PBPK simulator (Simcyp Ltd, Sheffield, UK) was used to generate concentration-time profiles of moxidectin and associated PK parameters. Clinical data from seven moxidectin pharmacokinetic studies, including a dose-ranging QTc study in healthy male subjects, which provided plasma concentration-time data across a broad range of doses [32], were used to develop and refine the PBPK model. Observed data from the dedicated breast milk excretion study [20] were used to verify the simulated plasma concentrations of moxidectin in healthy females prior to predicting exposures in neonates (0–1 month) and infants (1–6 months). Using the clinical lactation data, it was estimated that 19.5 μg of the 56.0 μg (total) was excreted in breast milk on Day 1, followed by daily amounts of 6.63, 4.22, 2.72, 2.42, 1.85 and 1.71 μg up to and including Day 7. Thereafter, from Days 8 to 14, about 1 μg moxidectin was excreted daily, from Days 15 to 21 about 0.6 μg and from days 22 to 28 about 0.4 μg. These values were used as daily doses to simulate the infant exposures.

It should be noted that as the relative contributions of clearance routes (CYP3A4 versus biliary) of moxidectin were not fully characterised, a range of ontogenies were assessed, with a slow ontogeny representing the worst-case scenario. For simulations in neonates (0–1 month) and infants (1–6 months), the default CYP3A4 ontogeny within the Simcyp Simulator was applied [33]. However, for the biliary clearance component, both slow and fast ontogenies were considered [34].

All input data and absorption/distribution modules used for simulations of plasma concentrations of moxidectin are shown in Table 1. Absorption from solution was integrated within the Advanced Dissolution Absorption and Metabolism (ADAM) module of the model. Protein binding was determined via rapid equilibrium dialysis (RED) with incorporation of a presaturation step, based on methods reported previously [35]. The effective permeability across the intestine was predicted using the log P value [36]. A retrograde model was used to extrapolate back from a clearance value based on clinical data to a CYP3A4-mediated metabolic component (represented by *in vitro* data) and a biliary clearance component in the liver [37]. The volume of distribution (Vss) and the tissue partition coefficients (P_t:p_) were predicted using the methods described by Sawada *et al*. [38] and Rodgers and Rowland [39].

**Table 1:** Input parameters for moxidectin PBPK model.

Parameter	Value	Source
**Physicochemical/binding properties** Molecular Weight (g/mol)	639.8	
clog P	5.67	Predicted
Compound Type	Neutral	
Blood to plasma ratio	1	Assumed
fu	0.00078	Measured [35]
**Absorption Model**	ADAM	
**Distribution Model**	Full PBPK	
Vss (L/kg)	16.85	Predicted [38]
Prediction of Pt:p	Method 2	Predicted [39]
Pt:p adipose	57.85	Fitted based on clinical data in healthy male subjects [32]
Kp Scalar	0.3	
**Clearance**		
CL/F (L/h)	2.0	Clinical data [32]
CLint (μL/min/mg protein)	517	Predicted from CL/F [32]
CYP3A4 (μl/min/mg protein)	36 (7% of clearance)	In vitro metabolism data [37]
Biliary (μl/min/mg protein)	481	

Abbreviations: fu–unbound fraction in plasma; ADAM–advanced dissolution absorption and metabolism; Vss–volume of distribution; Pt:p–partition coefficient for tissues; CL/F–oral clearance; CLint–metabolic intrinsic clearance

## Results

### Method 1: Empirical approach

In a non-clinical pre- and post-natal (PPN) toxicology study in rats (data on file) with daily oral (gavage) dosing of moxidectin (rather than a single dose as used in humans), the no-observed adverse effect level (NOAEL) for maternal toxicity and embryo toxicity was 0.5 mg/kg given daily. The mean moxidectin concentration in the male and female rat pup plasma following a maternal dose of 1.5 mg/kg given daily was 214 ± 91.6 ng/mL. Thus, assuming dose proportional pharmacokinetics [10], the mean moxidectin concentration in male and female pup plasma at the NOAEL of 0.5 mg/kg given daily was estimated to be 71.3 ng/mL.

**Fig 1:**
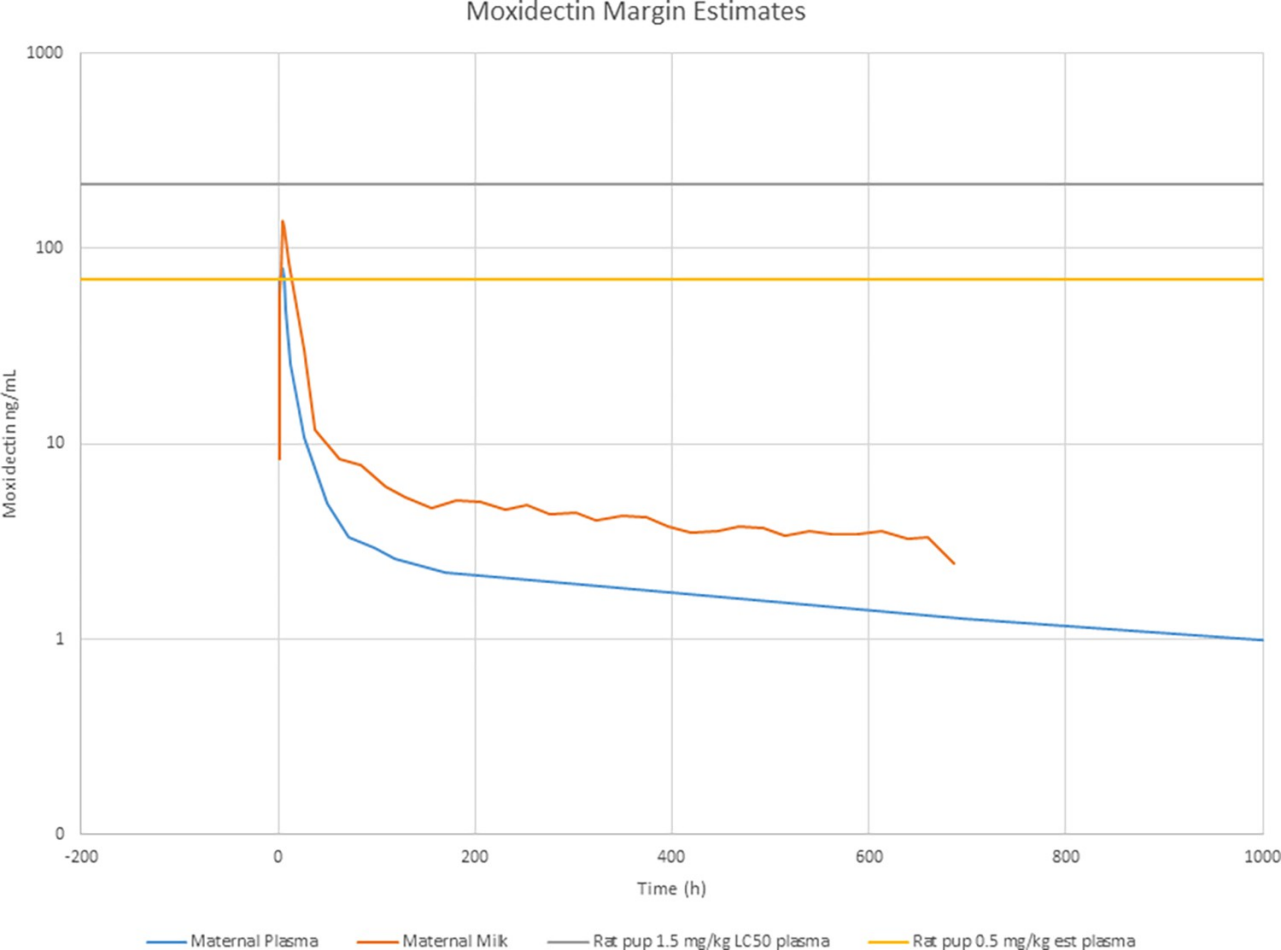
Mean (± SD) breast milk and plasma moxidectin concentration-time profiles in healthy lactating women after a single oral 8 mg dose (n = 12). The mean observed rat pup plasma level following the highest maternal dose of 1.5 mg/kg daily, and the mean estimated rat pup plasma levels at the NOAEL of 0.5 mg/kg daily in the non-clinical pre- and post-natal (PPN) toxicology study are also presented.

The mean moxidectin concentration-time profiles in plasma and breast milk from the regulatory-standard clinical PK study conducted in healthy lactating women are presented in Fig 1. The mean observed rat pup plasma level following the highest maternal dose of 1.5 mg/kg given daily in the non-clinical PPN study, and the mean estimated rat pup plasma level at the NOAEL of 0.5 mg/kg given daily are also presented in Fig 1.

In healthy lactating women, mean peak concentrations (C_max_) of moxidectin in both plasma and breast milk occurred at approximately 4 hours post dose followed by a rapid decline in moxidectin concentrations (Fig 1). Based on the NCA analysis of the plasma concentration-time data in healthy lactating women, the mean C_max_ of moxidectin was 87 ng/mL [20], approximately 2.5-fold lower than the observed rat pup plasma concentration of 214 ng/mL at the highest maternal dose in the PPN study, and approximately 1.2-fold higher than the estimated mean rat pup plasma concentration of 71.3 ng/mL at the NOAEL in the PPN study (Fig 1). By 24- and 48-hours post dose, the observed mean plasma concentrations of moxidectin in healthy lactating women were 10.7 and 5.1 ng/mL, respectively, approximately 7- and 14-fold lower than the estimated mean rat pup plasma concentration of 71.3 ng/mL at the NOAEL. At 1 week (168 hours) post dose, the observed mean plasma concentration of moxidectin in healthy lactating women was 2.2 ng/mL, approximately 32-fold lower than the rat pup plasma concentration at the NOAEL. A summary of the observed mean plasma concentrations of moxidectin derived by NCA at various timepoints up to 1 week after a single oral dose of 8 mg moxidectin to healthy lactating women compared to the estimated mean rat pup plasma concentration at the NOAEL in the non-clinical PPN study is presented in Table 2.

**Table 2:** Summary of mean observed plasma concentrations of moxidectin derived by NCA at 24, 48, 72 and 168 hours (1 Week) after a single oral dose of 8 mg in healthy lactating women compared to estimated mean rat pup plasma concentrations following 0.5 mg/kg/day maternal dosing in a pre- and postnatal (PPN) development toxicology study.

Timepoint (Hours post dose)	Human Lactation study Mean Maternal Observed plasma concentration (ng/mL)^a^	Rat PPN Study Mean Estimated Rat Pup plasma at NOAEL (ng/mL)^b^	Exposure Ratio Rat Pup: human
4 (C_max_)	87	71.3	0.8
24	10.7	71.3	6.6
48	5.1	71.3	14.0
72	3.4	71.3	22.2
168	2.2	71.3	32.4

^a^ Source: Mean observed plasma concentrations from data on file and Korth-Bradley [20]

^b^ Estimated assuming linear PK from mean rat pup plasma concentration of 214 ± 91.6 ng/mL at highest maternal dose of 1.5 mg/kg (PPN study, data on file). Rats dosed daily by oral gavage

Using the established pop PK model, the mean C_max_ for a typical healthy female with a bodyweight of 64 kg and a BMI of 22.8 kg/m^2^ (taken from Korth-Bradley et al [20]) was estimated to be 66.2 ng/mL following a single oral dose of 8 mg moxidectin. Mean plasma concentrations 3, 7 and 30 days after dosing were estimated to be 4.18, 2.61 and 1.57 ng/mL, respectively, based on the pop PK analysis. Similarly, in a typical onchocerciasis patient, mean C_max_ estimated using the pop PK model was 61.1 ng/mL with mean plasma concentrations of 4.64, 2.53 and 0.92 ng/mL on days 3, 7 and 30, respectively. The mean estimated plasma concentration data for onchocerciasis patients derived using the population PK model are similar to the observed mean values in heathy lactating females and are summarised at each of the various timepoints post dose in Table 3.

**Table 3:** Summary of mean estimated moxidectin plasma concentrations at C_max_, and at 3, 7 and 30 days in a typical healthy female subject^a^, a typical female onchocerciasis patient^a^ and mean observed moxidectin plasma concentrations in healthy lactating females^b^ after a single oral dose of 8 mg.

Estimated mean plasma concentration (ng/mL) ^a^			Observed mean plasma concentration (ng/mL)^b^
Sample Time	Healthy Female Subject	Female with Onchocerciasis	Healthy Lactating Females
C_max_	66.2	61.1	87
Day 3	4.18	4.64	3.4
Day 7	2.61	2.53	2.2
Day 30	1.57	0.92	1.3*

^a^ Estimated using an established population PK model parameterized using data from moxidectin clinical studies

^b^ Source: Korth-Bradley [20]. *plasma sample taken on Day 29 in healthy lactating females

The overall mean (± SD) plasma AUC in healthy lactating females following a single oral dose of 8 mg moxidectin was 4046 ± 1796 ng.h/mL. The mean plasma AUC at 1, 2, 3, 4, 8 and 12 weeks after dosing was determined using NCA, and the proportion of the overall mean plasma AUC accounted for within each time period was estimated to be 37.9, 46.8, 54.2, 60.3, 76.9 and 86.1%, respectively. Thus, based on these data, if plasma sampling had commenced 1 week after administration of a single oral dose of 8 mg moxidectin, the mean plasma AUC would have been approximately 62.1% of the overall observed plasma AUC in the regulatory-standard clinical study conducted in healthy lactating women. Similarly, if plasma sampling had commenced 4, 8 or 12 weeks (1, 2 or 3 months) after dosing, the mean plasma AUC’s would have been approximately 39.7%, 23.1% or 13.9% of the overall observed mean AUC, respectively.

The mean absolute infant dose, estimated as the amount of moxidectin excreted in milk following a single oral dose of 8 mg moxidectin, and based on the assumption that the infants would consume all of the milk collected was 56 ± 24 μg [20]. If milk collections had commenced 24 hours or 1 week after dosing, the mean absolute infant doses were estimated to be 40 ± 18 μg and 19 ± 10 μg, respectively.

### Method 2: PBPK approach

**Fig 2:**
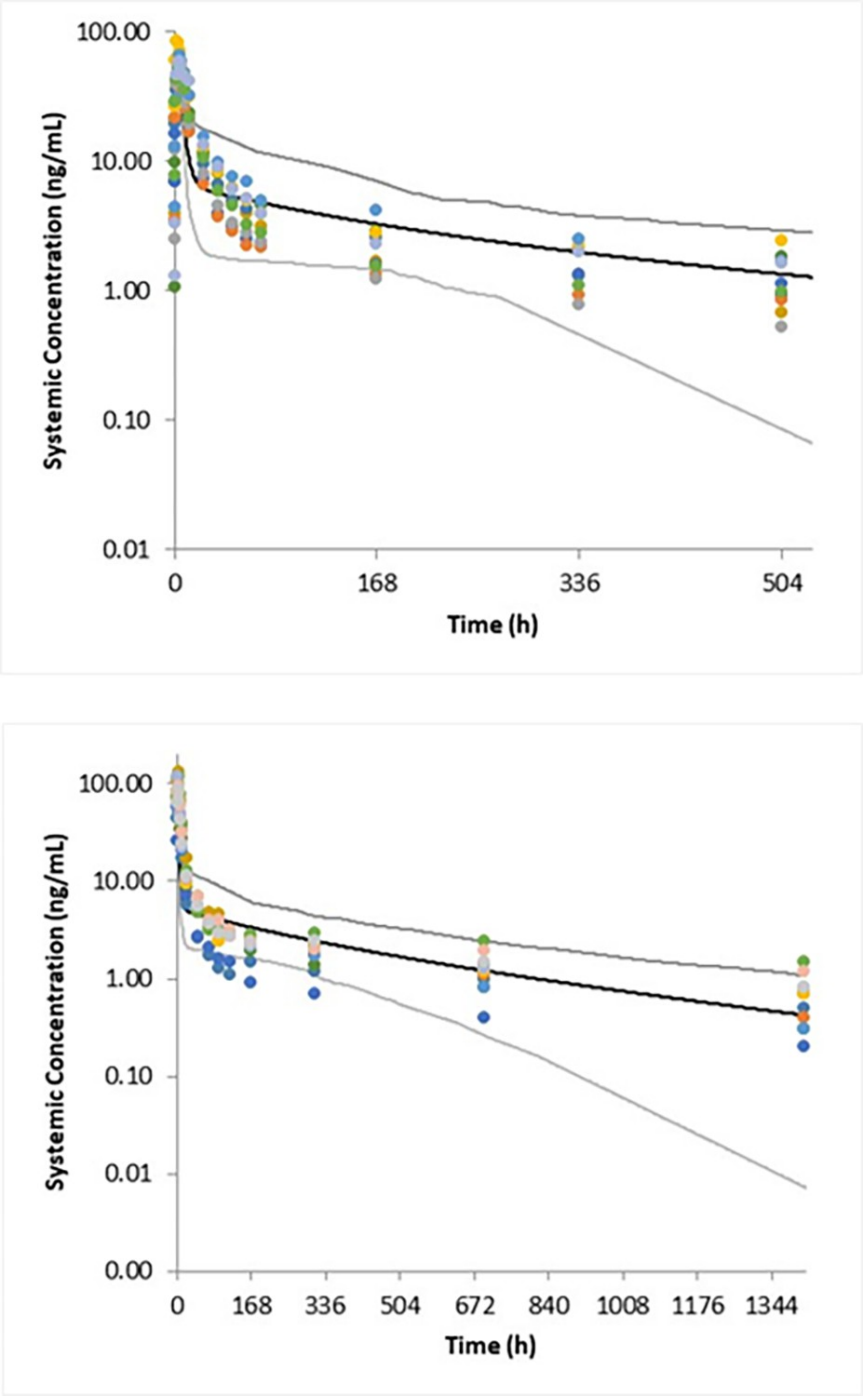
Simulation of plasma concentrations of moxidectin in healthy male subjects (top) and lactating women (bottom) after an oral dose of 8 mg using the PBPK model. Black and grey lines represent the mean and 5^th^ and 95^th^ percentiles of the virtual population; symbols represent the observed data [32, 20].

The PBPK model was able to capture the observed profiles of moxidectin with reasonable accuracy in both healthy male and healthy female subjects (Fig 2), predicting exposures of moxidectin that were within 1.3- to 1.5-fold of observed data.

**Fig 3:**
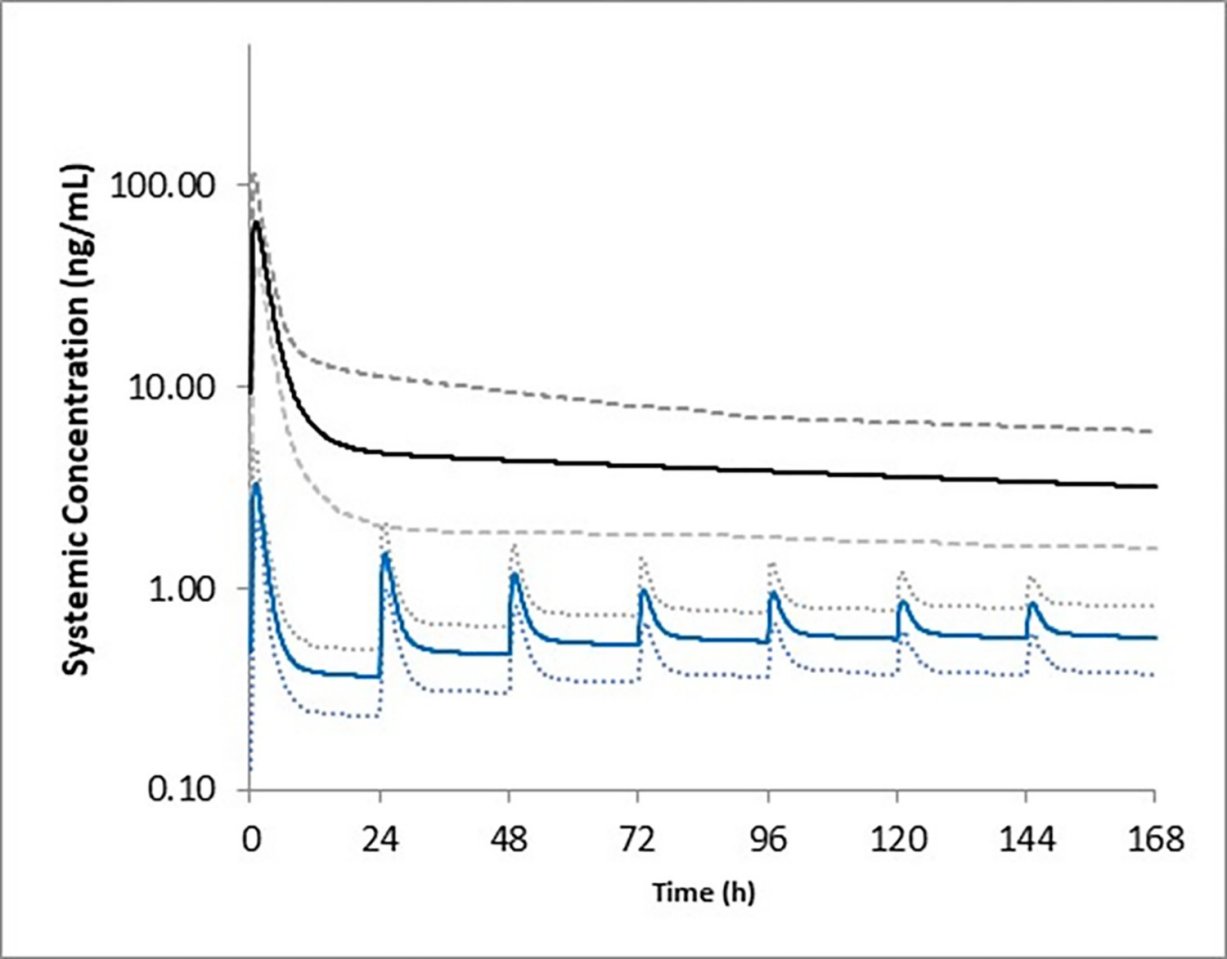
Predicted plasma exposures in infants (blue lines) during breastfeeding following administration of 8 mg moxidectin in the mothers (black lines). Lines represent the mean and 5^th^ and 95^th^ percentiles of the virtual populations. The estimated total daily amount of moxidectin excreted in breast milk was used as the daily dose to simulate infant exposures.

Simulations run in neonates and infants (up to 6 months old), where slow ontogenies were applied, indicated that on day 1, C_max_ was approximately 3.29 ng/mL (20-fold lower than in the mother; Fig 3) and after 4 weeks, plasma exposures were about 0.24 ng/mL.

**Fig 4:**
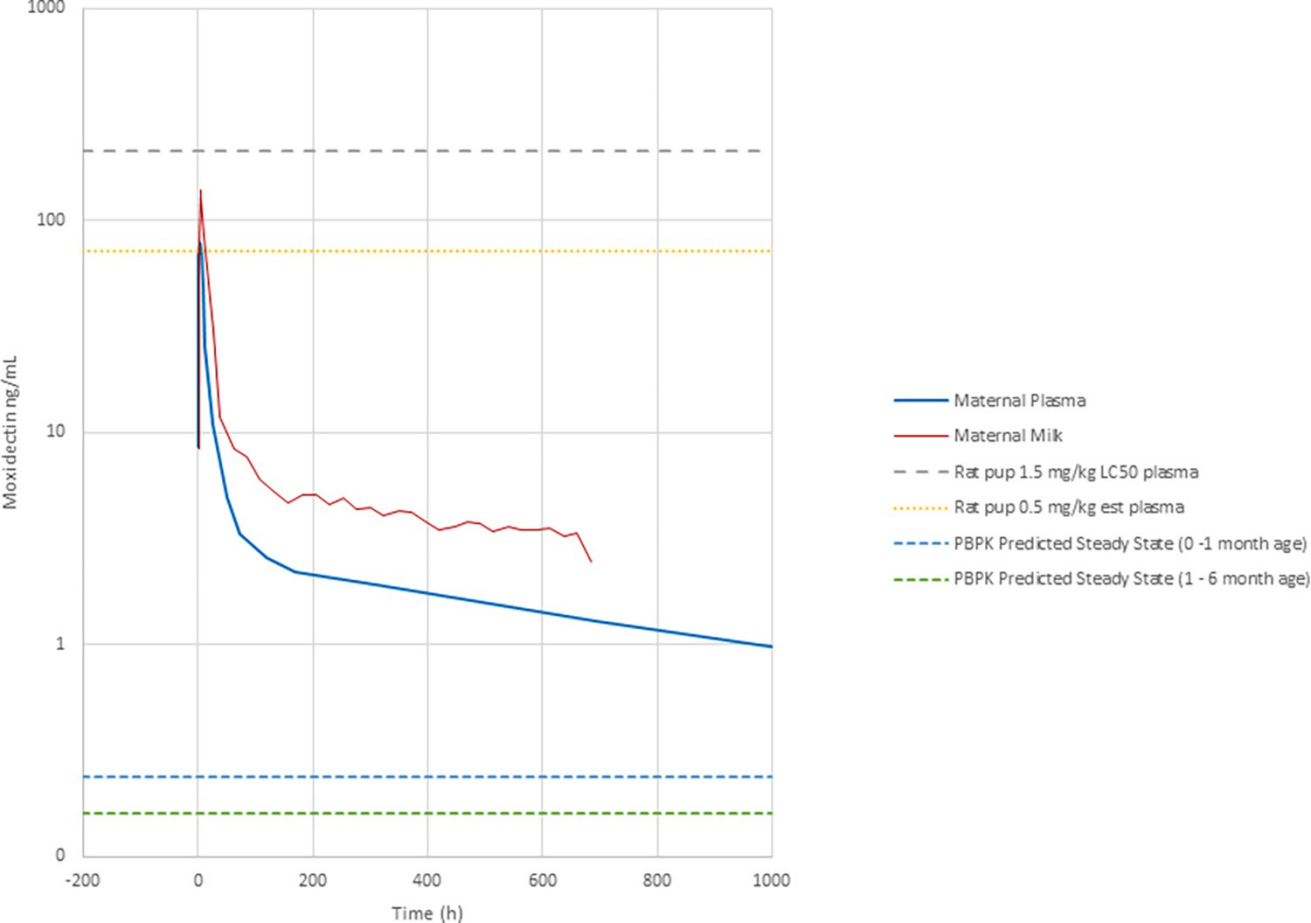
Graphical representation of moxidectin toxicokinetics margins based on rat pup LD10 concentrations versus PBPK-predicted steady state concentrations for infants aged 0 to 1 month and from 1 to 6 months. The mean observed rat pup plasma level following the highest maternal dose of 1.5 mg/kg daily, and the mean estimated rat pup plasma levels at the NOAEL of 0.5 mg/kg daily in the non-clinical pre- and post-natal (PPN) toxicology study are also presented.

When put into context of the mean plasma moxidectin concentration of 214 ng/mL in rat pups on lactation day (LD) 10 following pre-term exposure to maternal moxidectin (dosed at 1.5 mg/kg each day) and post-term exposure to moxidectin in rat milk (857 ng/mL) (data on file), a toxicokinetic margin of approximately 65x on Day 1 and approximately 3500x after 4 weeks in infants receiving moxidectin in milk as described above would be predicted (Fig 4).

## Discussion

The prescribing information contains the essential scientific information needed for safe and effective use of a pharmaceutical drug product and represents the pivotal source of data for healthcare professionals. However, consistent with the challenges and cost of regulatory product development, there is often a limited amount of data with respect to “special populations” in these documents, resulting in the prescribing community using available data and best estimates to guide treatment. Evaluation of drug concentrations in breast milk and the extent of potential exposure in breasfted infants provides valuable information for the generation of dosing recommendations. Although information on drug use during lactation is available through sites such as LactMed [40], the data available are often incomplete, including only lactation data from animal studies with little or no relevant published information on human maternal or infant levels. Furthermore, as the composition of milk is known to vary between species, with differences in protein and fat content [41], it has been suggested that animal models of drug excretion into milk may not be particularly useful to determine safety in humans. Anderson and Sauberan [42] concluded that common phrases in older product labelling that a drug is excreted into the milk of lactating animals may “essentially be irrelevant to drug safety in human lactation, because it is the quantity that is important, not the mere presence or absence of the drug in milk”. Developmental differences between species should also be taken into consideration when using animal models of drug excretion in milk to determine safety in humans. For example, the blood brain barrier in humans, dogs, cattle, sheep and other species is functional at birth, whereas in rodents the blood brain barrier is not functional until after postnatal day 14, and the multidrug efflux transporter P-glycoprotein (P-gp) is not expressed in the rodent until after postnatal day 10. Observed toxicities of some drug products in neonatal rats during the first postnatal week could therefore be due to a combination of excessive plasma levels, as a result of exposure through maternal milk, plus increased permeability of the blood-brain barrier due to a lack of P-gp. These data support the further conclusions of Anderson and Sauberan that only human studies can reliably quantify drug passage into breastmilk [42].

In April 2016, the US FDA sponsored a 2-day public workshop to discuss the safety of drugs and biological products used during human lactation [43] the aims of which were to provide a forum to discuss the collection of data, to inform potential risks to breastfed infants, and considerations for future approaches to design and guide clinical lactation studies. The workshop concluded that, for many drugs, there was a paucity of high-quality clinical data in the literature and in drug labelling about use in lactating women and their infants. The report also concluded that prospective studies measuring drug concentrations in human milk are necessary to establish an accurate risk-benefit assessment, which is further enhanced by measuring infant serum concentrations and infant clinical outcomes. It was also acknowledged that novel techniques such as PBPK and population PK modeling had been used for a few drugs, and, at the time of workshop, they were considered to represent promising future approaches to predict milk concentrations [43]. In the present study we have described two methods to estimate moxidectin exposure in breasfted infants using data from a regulatory-standard clinical study in which moxidectin plasma concentrations and excretion into breast milk were quantified following the recommended therapeutic dose of a single oral dose of 8 mg to healthy lactating women [20]. The methods used were in line with recommendations from the FDA workshop. For the empirical analyses, plasma concentration data and breast milk drug concentrations were derived from the regulatory-standard clinical study conducted in healthy lactating women, and the model-based simulations were validated using human exposure data from several clinical studies which provided plasma concentration-time data across a broad range of doses. In addition, the ontogeny of metabolizing enzymes was taken into account in the model-based simulations. The resulting estimated exposure data using both methods were then placed into perspective with regard to potential risks using moxidectin toxicology data and established moxidectin acceptable daily intake level recommendations based on exposure from animal food [23,24].

In the regulatory-standard clinical study conducted in healthy lactating women, concentrations of moxidectin in breast milk followed a similar pattern to those in plasma, with maximum concentrations (C_max_) occurring approximately 4 hours after dosing followed by a rapid decline (Fig 1). Although the observed mean C_max_ in both breast milk and plasma in healthy lactating women was slightly higher than the estimated mean plasma concentration in male and female rat pups at the NOAEL of 0.5mg/kg given daily in a non-clinical toxicology study, concentrations in both plasma and breast milk decreased to well below the estimated plasma concentration at the NOAEL within 24 hours following drug administration. One week after administration of the recommended therapeutic dose of 8 mg moxidectin to healthy lactating women, mean plasma concentrations were approximately 40-fold lower than the observed C_max_, and 32-fold lower than the estimated NOAEL in the non-clinical toxicology study. In addition, an assessment based on plasma AUC indicated a reduction of 39.7% in the observed systemic exposure to moxidectin in healthy lactating women the day after dosing. Based on these empirical analyses, a delay between the time of treatment and the onset of breastfeeding would therefore be expected to result in a considerable reduction in the exposure of moxidectin to the infant via breastmilk. These data were further supported by the population PK estimates of plasma concentrations in healthy lactating females and for a typical onchocerciasis patient. Pop PK derived estimated exposures at 3, 7 and 30 days after a single oral dose of 8 mg moxidectin were shown to be substantially lower than the peak plasma concentrations observed in the PPN rat study.

In addition to comparisons of the estimates of systemic exposures in healthy lactating women relative to the toxicology data, estimates of the absolute and relative infant dose (RID) were also taken into consideration. In the healthy lactating women study by Korth-Bradley [20], the RID was determined as the ratio of the absolute infant dose (normalized to an infant weight of 5 kg) to the maternal dose of 8 mg (normalized by each subject’s weight). The absolute infant dose was calculated as the sum of the individual milk volumes and concentrations in breast milk. The RID was estimated to be 8.7 ± 3.2%, which is below the value of 10% of a maternal dose that the WHO working group considers to be a generally acceptable level of infant drug exposure [42]. It should be noted that the RID determined in this study represents the most conservative estimate as it was calculated using the assumption that the infant would consume the whole of the breast milk collected over the entire 30-day milk collection period. Given the observed decline in breast milk levels of moxidectin after dosing (Fig 1), a hold on breastfeeding of as little as 24 hours following drug administration would result in a substantial decrease in the estimated absolute infant dose and hence the estimated RID would also be substantially lower.

The absolute infant dose in the same Korth-Bradley study was 56 ± 24 μg, with a range from of 32.7 to 110.2 μg [20]. These data represent the total amount of moxidectin excreted into breast milk during the complete milk collection period of 30 days. The average exposure to moxidectin per day in breast milk is therefore estimated to be approximately 1.1 to 3.7 μg, or 0.36 to 1.22 μg/kg/day based on a 3 kg (new-born) infant. Estimates of the amount of drug excreted in milk following a delay between dosing and the onset of breastfeeding indicated that there would be a corresponding reduction in the absolute infant dose. The toxicological acceptable daily intake levels for moxidectin based on secondary exposure from veterinary use established in Europe and the US are 3 μg/kg/day and 4 μg/kg/day per person, respectively [23,24]. Thus, based on the estimated absolute infant doses, it could be argued that no delay between treatment and the onset or resumption of breastfeeding would be necessary, as the estimated absolute infant doses are below the moxidectin acceptable daily intake levels in both the EU and US.

The conclusions of the empirical analyses were further supported by the PBPK analyses. Whereas the empirical analyses rely on inferred exposures in infants based on exposures in plasma and breast milk, the PBPK analyses provide more direct estimates of infant exposure. Furthermore, PBPK estimated infant exposures were assessed across the age range from neonates up to 6 months old, which is important considering the complex distribution of moxidectin and age-related changes in tissue volumes. In addition, whilst the empirical approach does not accommodate all prior information on both the drug and the physiology involved, a key advantage of the PBPK modeling is the ability to include sources of physiological and biochemical variability in the system parameters and to simulate the expected PK in a population of individuals rather than for an average subject [27,30]. However, in agreement with the recommendations of Anderson and Sauberan [42], and the US FDA workshop [43], both the PBPK and empirical analyses were based on the quantitation of drug in human breast milk in a regulated clinical study.

Being able to dose without regard to breastfeeding, or limiting the time between treatment and resumption of breastfeeding is an important medicine characteristic. Both the empirical and the PBPK analyses support the observation that no delay between moxidectin administration and the onset of breastfeeding would result in exposures to infants that are well below the exposures at the NOAEL level in the non-clinical toxicology study referenced. Infant exposures through breast milk for 1 to 7 days after maternal moxidectin dosing would also be well below both internationally established average daily intake (ADI) levels for moxidectin and the RID levels which the WHO working group considers to be acceptable. In summary, these quantitative empirical and model-based PBPK analyses supported the conclusion that moxidectin exposure to neonates and infants is significantly lower than the NOAEL observed in the non-clinical toxicology study. These results therefore highlight the benefit of adopting an integrated approach to generate dosing recommendations to support the safe and effective use of medicines. In the case of moxidectin, these data may be of benefit to policy makers regarding the timing of treatment of breastfeeding mothers who may be participating in MDA programs.
